# A Comparison of Physical Activity Between Home-Based and Centre-Based Pulmonary Rehabilitation: A Randomised Controlled Secondary Analysis

**DOI:** 10.3389/fresc.2021.743441

**Published:** 2021-10-26

**Authors:** Elizabeth J. Horton, Justina Ruksenaite, Katy Mitchell, Louise Sewell, Christopher Newby, Sally J. Singh

**Affiliations:** ^1^Faculty of Health and Life Sciences, School of Life Sciences, Coventry University, Coventry, United Kingdom; ^2^Department of Respiratory Medicine, University Hospitals of Leicester NHS Trust, Leicester, United Kingdom; ^3^Centre for Exercise and Rehabilitation Science, University Hospitals of Leicester NHS Trust, Leicester, United Kingdom; ^4^Faculty of Medicine and Health Sciences, University of Nottingham, Nottingham, United Kingdom

**Keywords:** pulmonary rehabilitation, physical activity, chronic obstructive pulmonary disease (COPD), exercise, step count, sedentary time

## Abstract

**Background:** Pulmonary rehabilitation (PR) is a highly effective intervention for individuals with chronic obstructive pulmonary disease (COPD). Physical activity (PA) has been shown to increase after a centre-based programme, yet it is not clear if a home-based programme can offer the same benefit. This study aimed to evaluate the effect of home-based PR compared with the centre-based PR on the PA levels post 7 weeks of PR and 6 months follow-up.

**Method:** In this study, 51 participants with COPD, of them, 36 (71%) men completed physical activity monitoring with a SenseWear Armband, at three time points (baseline, 7 weeks, and 6 months). The participants were randomly assigned to either centre-based supervised PR (*n* = 25; 69 ± 6 years; FEV_1_ 55 ± 20% predicted) or home-based PR (*n* = 26; 68 ± 7 years; FEV_1_ 42 ± 19% predicted) programmes lasting 7 weeks. The home-based programme includes one hospital visit, a self-management manual, and two telephone calls. The PA was measured as step count, time in moderate PA (3–6 metabolic equivalent of tasks [METs]) in bouts of more than 10 min and sedentary time (<2 METs).

**Results:** Home-based PR increased step count significantly more than the centre-based PR after 7 weeks (mean difference 1,463 steps: 95% *CI* 280–2,645, *p* = 0.02). There was no difference in time spent in moderate PA was observed (mean difference 62 min: 95% *CI* −56 to 248, *p* = 0.24). Sedentary behaviour was also significantly different between the centre and home-based groups. The home group spent 52 min less time sedentary compared with the centre-based (*CI* −106 to 2, *p* = 0.039). However, after 6 months, the step count and time spent in moderate PA returned to baseline in both the groups.

**Conclusion:** This study provides an important insight into the role of home-based PR which has the potential to be offered as an alternative to the centre-based PR. Understanding who may best respond from the centre or home-based PR warrants further exploration and how to maintain these initial benefits for the long-term.

**Trial Registry:** ISRCTN: No.: ISRCTN81189044; URL: isrctn.com.

## Introduction

The international guidelines support the effectiveness of pulmonary rehabilitation (PR) in the treatment and management of patients with chronic obstructive pulmonary disease (COPD) (1). However, few patients would benefit from receiving such treatment (2). Many individuals are unwilling or unable to attend the hospital-based programmes (3) and high dropout and low adherence rates (2) highlights the need for alternative models to the centre-based PR.

Physical activity (PA) is an important clinical outcome and has been associated with improved dyspnoea (4), exercise performance, and reduced muscle weakness (5), quality of life (6), mortality (7) and hospital admissions (8). Despite PR being highly effective at improving exercise performance (6), it has limited effect on how much patients with PA daily perform (9). Those studies which investigated PA post PR showed some improvement in the terms of step count, activity counts, and reduced sedentary time (10), whilst others did not find any significant difference (11). The challenge remains how best to change the behaviour of patients to be more physically active and maintain such behaviour long-term.

There is growing evidence that home-based PR in patients with COPD can be delivered as an alternative to the centre-based PR programme at increasing quality of life of the patients with COPD (12–16). Home-based programmes have the advantage of being easier to access. However, little is known about the impact of home-based PR on physical activity levels. Hollands et al. (15) study of home- vs. centre-based PR did show a short-term increase in moderate to vigorous physical activity (MVPA). Although 1-year later, both the groups had declined to the baseline levels. Another study also showed that home-based PR could increase daily step counts (17). However, there is a lack of more descriptive studies comparing the centre-based and home-based PR. As opposed to centre-based PR, the patients in their own home environment could more directly apply the programmes to activities of daily living and enhance the overall PA levels.

We have previously described a non-inferiority randomised trial comparing home-based rehabilitation facilitated by a manual (SPACE for COPD) vs. centre-based PR (14). A subgroup of patients from this trial was assessed for physical activity and here we describe the findings. We hypothesise that the home-based PR can effectively increase the daily physical activity in patients with COPD. This study aimed to assess the activity after 7 weeks of intervention and 6 months following up with either the centre- or home-based PR. We looked at daily step count, time spent in bouts, and time spent sedentary.

## Materials and Methods

### Study Design

This was a single blinded randomised controlled trial (RCT). We compared centre-based PR with a home-based PR programmed supported with the SPACE for COPD manual at 7 weeks and 6 months follow-up. The study took place between November 2007 and July 2012. All the participants gave written informed consent, and ethical approval for the study was granted by Leicestershire, Northamptonshire, and Rutland Regional Ethics Committee, reference 07/Q2501/6. Trial registration: ISRCTN81189044.

### Participants

The main trial recruited participants who were referred to PR, had a medical research council dyspnoea scale (MRC) between 2 and 5 and a minimum age of 18 years. The participants excluded from the study were those who would routinely miss the PR if they had significant neurological or locomotive disorders or unstable psychiatric history, and those who had participated in PR in the previous 12 months. In addition, those with poor English language skills were also excluded due to the manual only being available in English. The participants were randomised using the random block-sized sealed envelopes, into either conventional centre-based PR or home-based PR supported by the SPACE for COPD manual. The participants in the centre-based PR group enrolled on the two times weekly supervised exercise and education programme over 7 weeks. Those in the home-based group had an initial introductory session at the hospital led by a healthcare professional trained in motivational interviewing. This session introduced them to the manual and instructed them on how to complete their exercise. This group also received two telephone calls at 2 and 4 weeks to assess the progress, support, and motivation. It was anticipated that it would take to 7 weeks to progress through the SPACE for COPD manual, although it was their decision to keep supporting lifelong lifestyle change.

**Figure 1:**
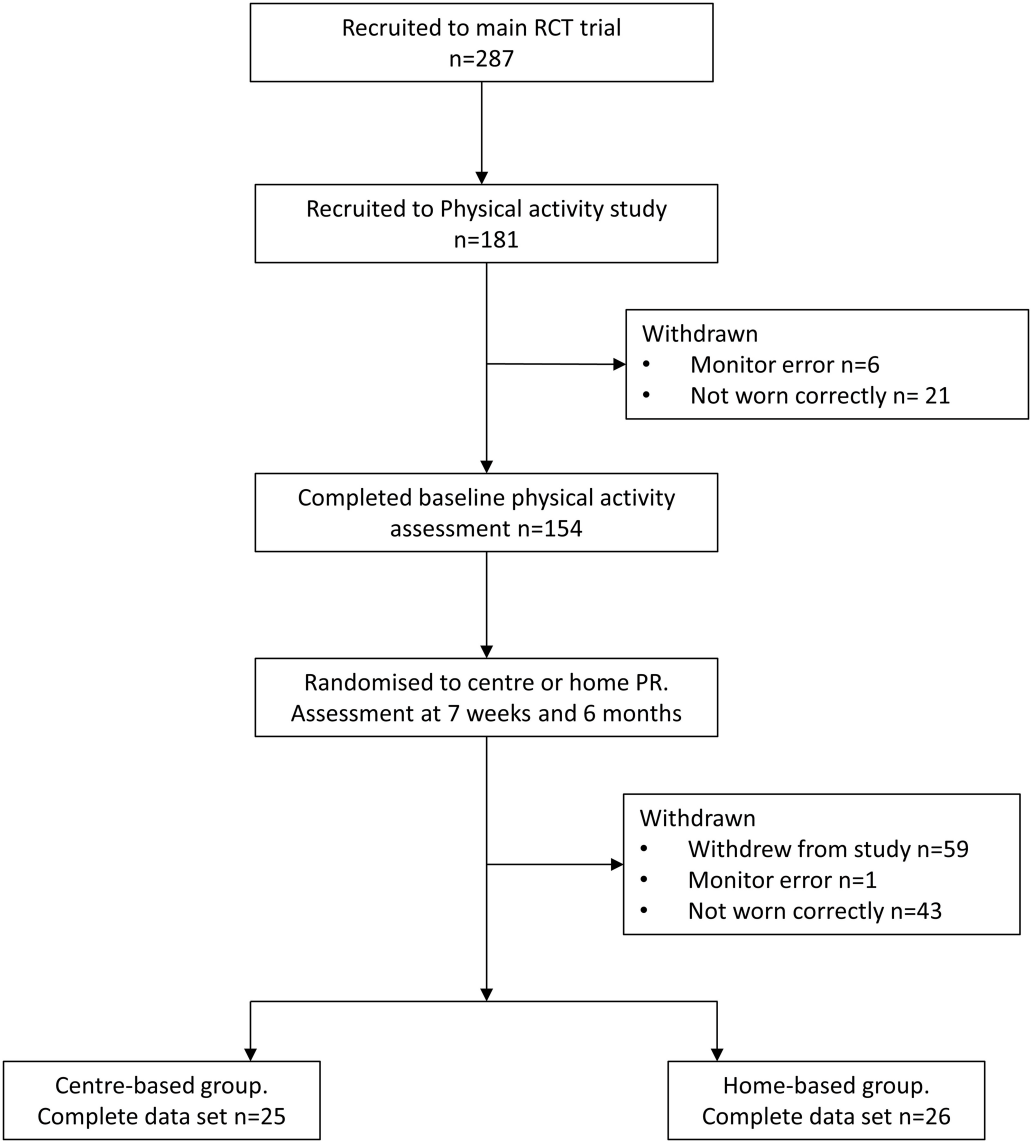
CONSORT flow of participants through the trial.

As part of the larger RCT comparing, the centre-, and home-based PR data were collected on the physical activity levels in a subgroup of the participants. Due to the limited number of activity monitors, the participants were recruited for this study based on the monitor availability. In total, 287 participants consented to the main trial and 154 provided the baseline physical activity data. The complete datasets, including baseline, 7 weeks, and 6 months, were available from 51 participants (Table 1). The flow of participants through the trial is presented in Figure 1.

**Table 1:** Mean (SD) characteristics of the centre-based and home-based groups who participated in the physical activity monitoring study.

	**Participant Characteristics**	
	**Centre-based (*n =* 55)**	**Home-based (*n =* 63)**
Age, yrs	67 (6.9)	67 (9.3)
Male:Female, *n*	37:18	43:20
BMI, m/kg^2^	27.88 (6.61)	26.80 (5.95)
FEV_1_, litres	1.37 (0.64)	1.19 (0.52)
FEV_1_ %predicted	51.43 (18.77)	47.22 (18.03)
FVC, litres	2.89 (0.98)	2.62 (0.91)
**MRC**, ***n*** **(%)**		
2	11 (20)	16 (25)
3	24 (44)	21 (33)
4	16 (29)	18 (29)
5	4 (7)	8 (13)
SpO_2_ rest %	90.1 (5.1)	90.6 (4.9)
**Smoking status**, ***n*** **(%)**		
Current smoker	10 (18)	16 (25)
Never smoked	2 (4)	7 (11)
Ex-smoker	43 (78)	40 (64)
Pack years	43.90 (26.47)	46.33 (38.01)
ISWT, m	287.09 (141.76)	276.98 (154.01)
ESWT, s	212.20 (122.81)	223.54 (222.03)

*Data presented as Mean and SD. BMI, body mass index; FEV_1_, forced expiratory volume in 1 s; FEV_1_% predicted, percentage of predicted forced expiratory volume in 1 s; FVC, forced vital capacity; MRC medical research council dyspnoea scale; SpO_2_%, percentage oxygen saturation; ISWT, incremental shuttle walk test; ESWT, endurance shuttle walk test*.

### Outcome Measures and Intervention

The physical activity was measured using the SenseWear armband (SWM; BodyMedia, Pittsburgh, PA, USA) and the participants were asked to wear the monitor for 5 days (including 2 weekend days) for all waking hours, apart from whilst bathing, showering, or swimming. The data from the SWM were downloaded using the InnerView™ software. The outcome measures at 7 weeks and 6 months were daily stepping count, time spent in moderate physical activity (>3 metabolic equivalent of tasks [METs]) in ≥10 min bouts, and time spent sedentary. The outcome measures at the baseline level were step count and time in moderate physical activity (>3 METs) in ≥10 min bouts.

The data were excluded from the analysis for the following reasons: if an error message occurred when downloading the SWM data, days where the data were <12 h, or if the monitor was worn for fewer than 3 days.

### Data Analysis

The data were analysed for normality. The paired *t*-test was used to analyse within the group differences from the baseline to 7 weeks and an independent *t*-test to determine the differences between the groups. Between group differences over time to 6 months were analysed using an ANOVA. The data processing and analysis were undertaken using IBM SPSS statistics 20.

## Results

In this study, 154 patients took part in baseline PA monitoring, 59 withdrew from the main trial, and the findings from 43 monitors were discarded as they were not worn for at least 12 h on at least 3 days. One set of data was discarded due to an error message on the SWM. This left 51 subjects with a minimum of 3 days of 12 h data at each of the time points (baseline, 7 weeks, and 6 months). Therefore, 25 patients were analysed in the centre-based group, 26 patients were analysed in the home-based group. A significant difference in the percentage of predicted FEV_1_ was detected at the baseline between the groups, but no other significant differences (Table 1).

### Intervention Effects

**Figure 2:**
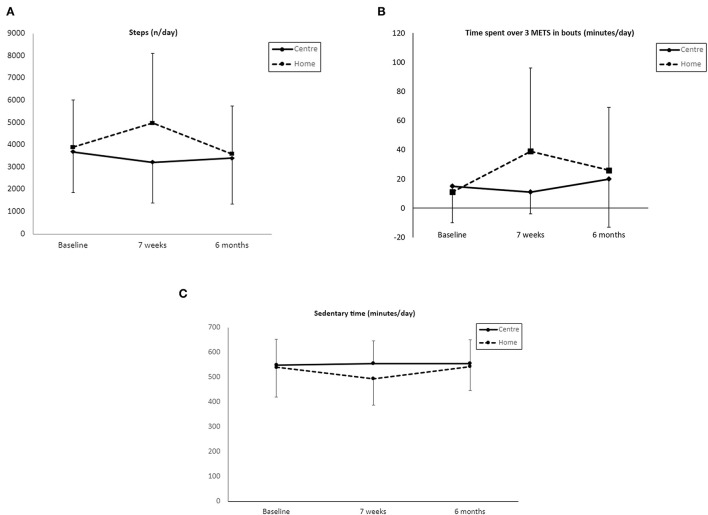
Baseline, after 7-week intervention and 6 months mean scores (± SD) in, **(A)** step count; **(B)** time spent over 3 METS in bouts; **(C)** sedentary time.

The results show that at 7 weeks, there were significant differences between the centre-based and home-based groups in the number of steps and time spent over 3 METs in bouts (Table 2). With a between-group difference of 1,463 steps (95% *CI* 280–2,645, *p* = 0.020) and 32 min longer time spent over 3 METS in the bouts (95% *CI* 11–54, *p* = 0.006), respectively, in favour of the home-based group. Within a home-based group, step count and time over 3 METs in the bouts increased significantly from the baseline by 1,074 steps (95% *CI* 289–1,708, *p* < 0.008) and by 28 min (95% *CI* 9–48, *p* < 0.008), respectively. No significant change was found in the PR group (Figures 2A,B). However, at 6 months, there was no significant difference between the centre-based group and home-based group even though time over 3 METs in the bouts remained improved in the home-based group. At 6 months, the home-based group spent 15 min more in time over 3 METs in the bouts (*p* = 0.037) whilst centre-based patients only 5 min (Figure 2B). In terms of step count, intergroup difference of 185 steps (95% *CI* 1,004–1,374) was not significant, and it went back to baseline within the groups (Figure 2B).

**Table 2:** Physical activity outcomes.

	**Within group difference**				**Between group differences**	
	**Home based group (*****n =*** **26)**		**Centre based group (*****n =*** **25)**		**Home-centre (*****n =*** **51)**	
	**Mean (95% CI)**	**Mean (95% CI)**	**Mean (95% CI)**	**Mean (95% CI)**	**Mean (95% CI)**	**Mean (95% CI)**
	7 weeks	6 months	7 weeks	6 months	7 weeks	6 months
Steps/day	1,074 (289–1,708)^*^	−309 (−1,211 to 230)	−464 (−1,445 to 517)	−275 (−852 to 697)	1,463 (280–2,645)	−34 (−1,062 to 994)
Sedentary time, min/day	−46 (−86 to −11)^*^	3 (−18 to 42)	6 (−38 to 45)	7 (−18 to 31)	−52 (−106 to 2)^**^	−4 (−40 to 32)
LPA, min/day	24 (−10 to 45)	−16 (−39 to 16)	6 (−23 to 33)	−15 (−33 to 16)	13 (−26 to 51)	−1 (−35 to 33)
MVPA, min/day	18 (−5 to 42)	−5 (−37 to 6)	−8 (−28 to 73)	−5 (−25 to 10)	62 (−56 to 248)	0 (−27 to 26)
VPA, min/day	4 (−4 to 14)	4 (−0.5 to 3)	−4 (−15 to 7)	1 (−17 to 12)	9 (−5 to 23)	3 (−12 to 19)
EE over 3 METs, min/day	83 (−124 to 288)	−48 (−143 to 46)	−94 (−61 to 63)	−53 (−253 to 148)	181 (−74 to 436)	5 (−213 to 222)
Time over 3 METs in bouts, min/day	28 (9 to 48)^*^	16 (−4 to 27)	−4 (−13 to 6)	5 (−11 to 13)	32 (11 to 54)	11 (−10 to 31)

*Data are means and 95% CI*.

* *p < 0.05 compared with baseline*.

** *p < 0.05 compared between the groups. LPA, light physical activity; MVPA, moderate to vigorous physical activity; VPA, vigorous physical activity; EE, energy expenditure; Bout activity lasting at least 10 min*.

Sedentary behaviour was also significantly different between the centre and home-based groups. The home group spent 52 min less time sedentary compared with the centre-based (95% CI −106 to 2, p = 0.039; Figure 2C). Within group difference following home-based PR at 7 weeks was 46 min less time spent sedentary (95% CI −86 to −11, p = 0.013), whilst centre-based PR spent only 6 min less time sedentary. Time spent sedentary was replaced by light PA and MVPA activity at 7 weeks. However, despite this numerical improvement, the latter two parameters did not reach statistical significance.

At 6 months, both the centre-and home-based groups' sedentary behaviour went back to the baseline and the intergroup difference was not significant.

## Discussion

There is a paucity of data exploring the home-based PR on PA. Compared with the previous studies, this data demonstrates the importance of group differences. It identified that home based programme was superior to usual centre-based PR at 7 weeks for increasing the step count exceeding the MCID proposed by Demeyer et al. (18) of 600–1,100 steps, time spent over 3 METs and reducing sedentary time. However, at 6 months postintervention, there was no significant difference in change between the two groups, even though the home-based patients with PR had significantly increased time spent over 3 METs and reduced sedentary time whilst the centre-based patients with PR did not. This suggests that the highly structured workbook and support from healthcare practitioners can produce a meaningful change.

We did not detect any improvement in the physical activity levels in the PR group. There is variability in the reports from other studies with an overall small increase in PA. Some authors suggested a longer time is required (9). PR focuses on the completion of bouts of walking, thus it is surprising that the patients did not improve on this aspect. On the other hand, it is possible that those in the PR group abdicated responsibility for their health to the healthcare team and relied purely on their supervised exercise sessions. Therefore, they did not perform more exercise at home.

At 6 months after the centre-based and home-based PR activity levels returned to baseline. The lack of long-term effects post PR was an expected finding: it is an established problem (19), and this study shows that home-based PR is not superior in this respect. We anticipated that within their home environment, the patients were more likely to maintain exercise behaviour. On the other hand, even though the differences were not significant between the two programmes, 6 months post home-based PR time spent over 3 METS was significantly higher compared with the baseline. This positive trend could be explored further. The optimal duration of the programmes is still not clear (6). It has been previously demonstrated that a 6-month programme is required to alter the free-living activity levels of the patients (10).

The focus of PR has conventionally been to improve exercise capacity (1, 20). However, there is an increasing focus on PA as an outcome measure for PR which depend on how PA is reported may not directly align with the aims of PR. PR has the explicit aim of increasing MVPA, which may not be reflected in steps. It is interesting that the home-based intervention seems to produce greater improvements in both the MVPA and steps at 7 weeks. In addition, the intervention produced important changes in the exercise capacity and health-related quality of life exceeding reported MCIDs for the Chronic Respiratory Disease Questionnaire (CRQ)-dyspnoea score and the endurance shuttle walk test (ESWT), suggesting an effective alternative to conventional PR.

Effing reported that their home-based trial which measured PA showed that over 11-month intervention, there was a mean improvement of 1,190 steps per day (17). This is comparable with our home-based programme after 7 weeks. In a similar study, Holland et al. (15) demonstrated an 8-week improvement in the time spent in MVPA, with no differences identified in any other physical activity parameter and no difference between the groups. Compare with our data that found an increase in step count in the home-based group of 1,074 steps, which was significantly higher than the centre-based group and met the MCID (18). The PA data from our trial are more descriptive in determining the patterns and intensities of PA whilst step count provides limited information and therefore makes it difficult to determine if the guidelines have been met. Sedentary behaviour is a problem of its own (21), thus the reduction in time sedentary is an important achievement too. It was replaced by light physical activity which might be a more realistic target in the patients with COPD rather than vigorous activity. Although at 6 months overall PA levels have returned to the baseline, an increase in time spent in continuous bouts of the exercise was maintained.

The baseline data revealed significant differences in the bout and non-bout activity: when measured in bouts percentage who met guidelines drops to 18% from 67%. However, Donaire-Gonzalez (22) in a study, found that 61% of the patients fulfilled the recommended PA guidelines by performing moderate to vigorous (METs >2.6 and >3) activity in bouts. The discrepancy between our findings and Donaire-Gonzalez is difficult to explain since our study populations were similar in age and disease severity. Spanish population performs the same amount of exercise as the British population (23). It could be explained by seasonal differences as described previously (24). With more severe disease, the patients reduced duration and frequency of bouts (22).

The main limitation of this study is a limited sample size. Regarding the baseline PA analysis, data from the current study may not truly reflect whether guidelines have been met as SWM was not worn for a full week thus it might be difficult to assess compliance with the guidelines which are set for 5 days. The data were collected only for 12 h a day which might have missed periods of significant activity. The participants in the home group had a significantly lower FEV_1_% predicted which may also be considered a limitation to the study.

## Conclusion

This study provides an important insight into a structured home-based PR programme effect on the physical activity levels. It is an important finding since physical activity plays a key role in the management of COPD. The challenge remains in sustaining positive PA and exercise behaviour in this population.

## Data Availability Statement

The raw data supporting the conclusions of this article will be made available by the authors, without undue reservation.

## Ethics Statement

The studies involving human participants were reviewed and approved by Leicestershire, Northamptonshire, and Rutland Regional Ethics Committee. The patients/participants provided their written informed consent to participate in this study.

## Author Contributions

EH, LS, and SS designed the study and intervention. EH, LS, and KM completed the intervention and data collection. EH and CN analysed the data. EH, JR, and SS wrote the manuscript. All authors contributed to the article and approved the submitted version.

## Conflict of Interest

The authors declare that the research was conducted in the absence of any commercial or financial relationships that could be construed as a potential conflict of interest.

## Publisher's Note

All claims expressed in this article are solely those of the authors and do not necessarily represent those of their affiliated organizations, or those of the publisher, the editors and the reviewers. Any product that may be evaluated in this article, or claim that may be made by its manufacturer, is not guaranteed or endorsed by the publisher.
